# A longitudinal study of antibody responses to endemic HCoV and novel SARS-CoV-2 among mother-child pairs in Zambia

**DOI:** 10.1186/s12879-025-11974-4

**Published:** 2025-11-18

**Authors:** Natasha Makabilo Laban, Martin Rhys Goodier, Roma Chilengi, Harriet Ng’ombe, Adriace Chauwa, Samuel Bosomprah

**Affiliations:** 1 Basic Science and Immunology Department, Centre for Infectious Disease Research in Zambia, Lusaka, Zambia; 2 Department of Infection Biology, Faculty of Infectious and Tropical Diseases, London School of Hygiene and Tropical Medicine, London, UK; 3 Flow Cytometry and Immunology Facility, Medical Research Council Unit, The Gambia at London School of Hygiene and Tropical Medicine, London, UK; 4 Department of Biostatistics, School of Public Health, University of Ghana, Accra, Ghana

**Keywords:** Coronavirus, SARS-CoV-2, HCoV, Antibody, Child, Zambia

## Abstract

**Background:**

Seroprevalence estimates of endemic human coronaviruses (HCoV) and novel severe acute respiratory syndrome coronavirus 2 (SARS-CoV-2) are limited in Zambia. Information on development of acquired immunity to endemic HCoV in early life is also scarce and the potential cross-protective effect of HCoV immunity on SARS-CoV-2 remains debatable. We investigated seroprevalence of endemic HCoV in mother-child dyads and SARS-CoV-2 in children and explored the association between HCoV and SARS-CoV-2 antibodies in children to elucidate coronavirus seroepidemiology in Zambia.

**Methods:**

We measured endemic HCoV NL63, 229E, OC43 and HKU1 and SARS-CoV-2 Spike protein subunit 1 (S1) specific immunoglobulin G (IgG) using an immunoassay. We tested plasma samples collected from Zambian mother-child dyads before the coronavirus disease 2019 pandemic in 2018 and 2019, and during the pandemic in 2020 and 2021. We determined HCoV S1 IgG seropositivity in mothers and children and SARS-CoV-2 S1 IgG seropositivity in children. We correlated HCoV antibodies in the mother-child pairs and longitudinally profiled HCoV antibodies in children to investigate development of HCoV immunity and contribution of maternal immunity. We compared child HCoV S1 IgG and SARS-CoV-2 S1 IgG antibodies before and during the pandemic to explore cross-reactivity.

**Results:**

HCoV and SARS-CoV-2 S1 IgG antibodies were detected among mothers and children. Child HCoV seroconversion occurred following waning of maternal immunity but there was no significant correlation between HCoV and SARS-CoV-2 S1 IgG before and during the pandemic. A rise in SARS-CoV-2 S1 IgG seroprevalence among children was observed following the second and third epidemic waves of the COVID-19 pandemic in Zambia.

**Conclusions:**

Endemic HCoV NL63, 229E, OC43 and HKU1 widely circulated and are acquired early in Zambia. SARS-CoV-2 seroprevalence in children speaks to the susceptibility of this population to infection that necessitates their inclusion in control measures.

**Supplementary Information:**

The online version contains supplementary material available at 10.1186/s12879-025-11974-4.

## Background

Human coronaviruses (HCoV) are a significant cause of respiratory illness in humans, particularly in children, and primarily associated with upper respiratory infections and gastrointestinal disease [1, 2]. Approximately 4–6% of children with acute respiratory illness are infected with endemic HCoV such as the Alphacoronaviruses HCoV NL63 and 229E and the Betacoronaviruses HCoV OC43 and HKU1, often as co-infections with other respiratory pathogens [3–6]. In more recent years, highly pathogenic Betacoronaviruses namely severe acute respiratory syndrome coronavirus (SARS-CoV), middle eastern respiratory syndrome coronavirus (MERS-CoV) and the novel severe acute respiratory syndrome coronavirus 2 (SARS-CoV-2) have emerged and are associated with severe and highly fatal respiratory disease [2, 7]. SARS-CoV-2 which causes coronavirus disease 2019 (COVID-19) has been responsible for over 7 million deaths globally to date and remains a significant threat to public health [8].

Serology-based surveillance is a valuable tool for estimating population level immunity and understanding disease transmission dynamics which can inform public health control measures. In Zambia, the first SARS-CoV-2 cases were detected in March 2020 and since then there have been four COVID-19 epidemic waves through to December 2022 [9] but limited SARS-CoV-2 serological studies have been done in our setting. A pooled SARS-CoV-2 IgG seroprevalence of 10.6% was documented in an extensive study done across six districts in Zambia conducted in July 2020 across all ages during the first COVID-19 wave. A peri-urban community based study among individuals >15 years old reported a 13.5% SARS-CoV-2 IgG seroprevalence between November 2020 to February 2021 coinciding with the second wave [10]. Following the third and fourth waves between September 2021 and September 2022, and after the rollout of COVID-19 vaccines, a serosurvey among pregnant women aged 15–49 years attending antenatal care services in four districts reported overall cumulative 63.8% SARS-CoV-2 IgG seroprevalence [11]. Children are a susceptible sub-population and potential reservoir of SARS-CoV-2 transmission particularly within households [12]. Children who often show milder SARS-CoV-2 infections compared to adults have thus far been generally excluded from vaccination strategies and SARS-CoV-2 seroprevalence studies are limited in this population but can be crucial in enhancing control strategies [13–16]. As of July 2022, only 28 studies within the entire African region had reported SARS-CoV-2 seroprevalence estimates among children [17] and thus more studies are needed.

A pertinent research area to understand SARS-CoV-2 epidemiology is the immune response to endemic HCoV due to the arguable yet potential cross-reactivity with SARS-CoV-2. Endemic HCoV infections elicit robust IgG antibody responses [18] and prior exposure to endemic HCoV has been shown to influence the immune response to SARS-CoV-2 and alter the course of clinical presentation in children [19]. The coronavirus structural spike (S) protein is the major inducer of the host neutralizing antibodies targeted against its subunit 1 (S1) that mediates viral attachment and the subunit 2 (S2) involved in fusion and entry during infection [20, 21]. It has been shown that Betacoronaviruses (OC43, HKU1, MERS-CoV, SARS-CoV and SARS-CoV-2) and Alphacoronaviruses (NL63 and 229E) exhibit higher viral sequence homology within their respective genera than across them [22, 23]. This pattern also applies to T-cell epitopes, with HCoV Betacoronaviruses showing greater homology with SARS-Co-V-2 than Alphacoronaviruses [22]. Consequently, natural and experimental studies have shown stronger antibody reactivity within each genus rather than between them [18, 23]. Other studies demonstrate boosted antibody responses to the endemic HCoV Betacoronaviruses after SARS-CoV-2 infection and cross-reactivity against SARS-CoV-2 in pre-pandemic sera [24–26]. Understanding endemic HCoV seroprevalence can therefore provide additional insights towards observed population level SARS-CoV-2 immune profiles with important implications for COVID-19 vaccination strategies [27]. To the best of our knowledge no study has longitudinally profiled endemic HCoV serological responses and association with SARS-CoV-2 immunity in Zambian children with the only identified study having been conducted on adult samples [26].

The aim of this study was to investigate the seroprevalence of endemic HCoV and SARS-CoV-2 in Zambia to contribute to existing knowledge locally. The rationale to study mother-child pairs was to enable the investigation of the development of aquired endemic HCoV immunity, estimation of SARS-CoV-2 seroprevalence in an understudied child population after multiple COVID-19 epidemic waves and an exploration of the relationship between HCoV and SARS-CoV-2 immunity in an unvaccinated sub-population with roles in SARS-CoV-2 transmission. Results from this study can improve understanding of coronavirus immunity in children and the Zambian population.

## Methods

### Study design and participants

This was a longitudinal study nested under an existing rotavirus vaccine clinical trial detailed elsewhere [28]. Briefly, the trial was conducted at George Health Centre, a government run health facility serving a peri-urban community in Lusaka Zambia, and recruited mother-infant pairs from a population attending the health facility for routine vaccination visits as per the national immunisation schedule. The trial enrolled mothers and their children aged 6–12 weeks old (*n* = 214) who were followed up to when the child was 36 months old between September 2018 and November 2021. Inclusion criteria for enrolment was the mothers’ willingness to participate voluntarily and provide written informed consent, the child’s’ eligibility for oral rotavirus vaccination as per national policy, mothers’ willingness for herself and the child to undergo all study procedures and residence in the study area for the study duration. Children with contraindication to rotavirus vaccination, previous receipt of rotavirus vaccine, recent immunosuppressive therapy including high-dose systemic corticosteroids, history of receiving blood transfusion or blood products, including immunoglobulins within the previous 6 months, any condition deemed by the study investigator to pose potential harm to the child or jeopardize the validity of study result and any existing congenital anomalies were excluded. Plasma from whole blood samples collected from mothers and their children at enrollment and from children at scheduled visits over the follow up period was processed and stored at -20 degrees Celsius at the Centre for Infectious Disease Research in Zambia laboratory. The trial was registered under the Pan African Clinical Trials Registry (Reference number PACTR201804003096919) on 16th February 2018.

**Fig. 1:**
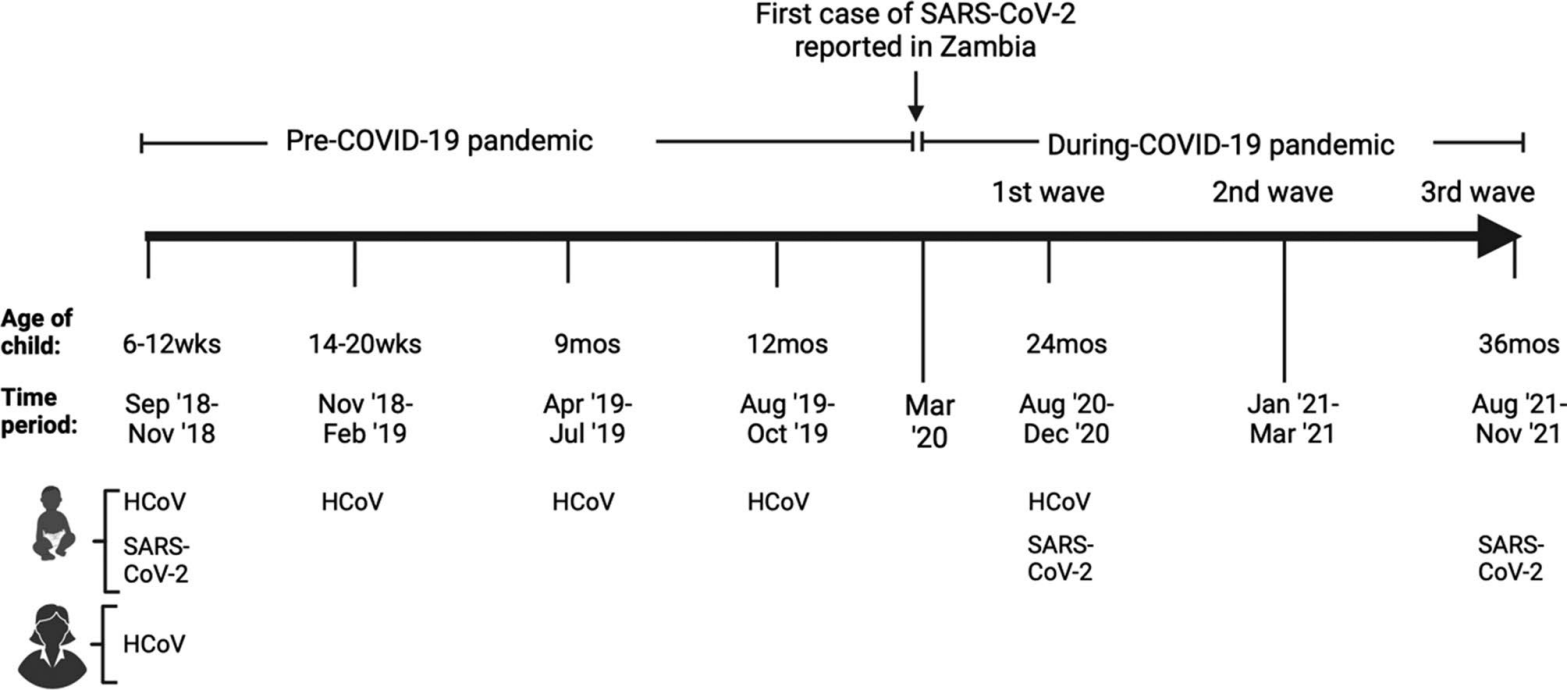
Study sampling schema. Illustration of the timepoints of plasma sampling from mothers and children under the parent rotavirus trial that were utilised for the nested longitudinal study. Created with BioRender.com

For this nested study, we made use of available participant data and stored plasma from both mothers and children collected before the COVID-19 pandemic between September 2018 and October 2019 and during the first three COVID-19 waves. In Zambia, the first wave of COVID-19 occurred from 1st June 2020 to 1st October 2020 followed by the second wave from 3rd January 2021 to 17th March 2021 driven by the beta B.1.351 SARS-CoV-2 variant, and the third wave from 29th May 2021 to 20th August 2021 driven by the Delta SARS-CoV-2 variant [9, 29, 30]. During the first wave, a partial lockdown and mandated community, health facility and healthcare worker and point of entry screening and contact tracing to identify cases and quarantine and isolation protocols were utilised for control [31]. Mandatory face masks, hand hygiene, physical and social distancing, restriction of gatherings and mass testing were also implemented [30]. Preventive measures during the second and third COVID-19 waves were similar to those implemented in the first wave but with added administration of COVID-19 vaccinations, temporary closure of learning institutions and specific retailers and enhanced surveillance, case management, COVID-19 communication and community engagement [30]. As illustrated in Fig. 1, plasma samples used in this study were collected from mothers and children before the COVID-19 pandemic when the child was 6–12 weeks old between September 2018 and November 2018 (baseline) and from children during scheduled visits when the child was 14–20 weeks old (November 2018-February 2019), 9 months old (April 2019-July 2019) and 12 months old (August 2019-October 2019). Plasma was also collected from children during the first COVID-19 wave at 24 months old (August 2020-December 2020) and the third COVID-19 wave at 36 months old (August 2021-November 2021).

**Fig. 2:**
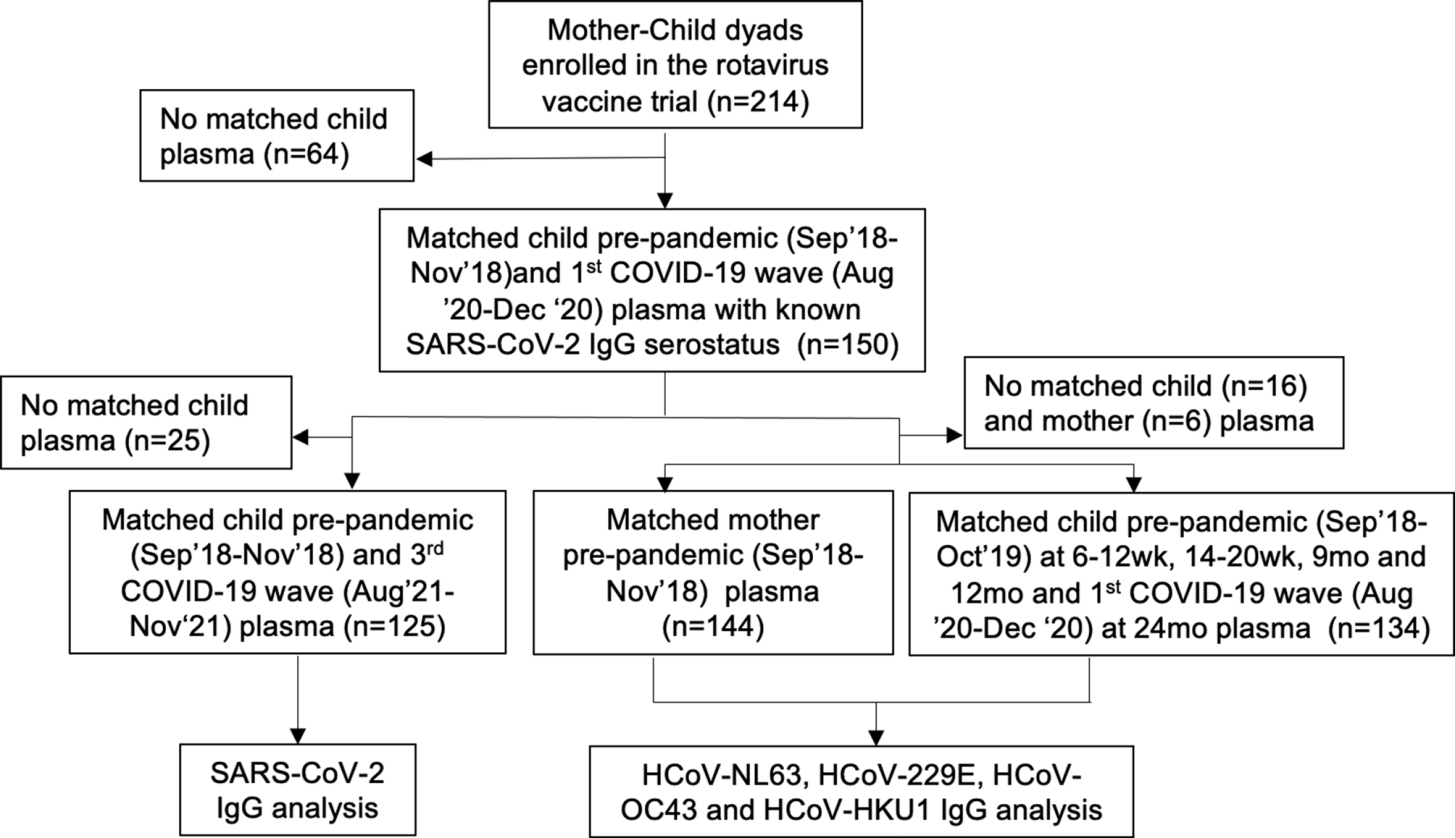
Participant flow chart. Illustration of the selection process for participants included in the study and plasma used in the laboratory analysis from a population of mother-child pairs enrolled in the parent rotavirus vaccine clinical trial

As shown in Fig. 2, we included all children with known SARS-CoV-2 S1 IgG serostatus (*n* = 150), measured in plasma collected between August 2020 to December 2020 during the first epidemic wave of COVID-19 pandemic in Zambia [9], as we have published elsewhere [32] and their mothers in this nested study for the measurement of endemic HCoV-NL63, HCoV-229E, HCoV-OC43 and HCoV-HKU1 S1 IgG antibodies. Among the 150 children, 148 (98.7%), 142 (94.7%), 145 (96.7%), 143 (95.3%), and 146 (97.3%) had sufficient plasma available for HCoV IgG testing at ages 6–12 weeks, 14–20 weeks, 9, 12 and 24 months respectively. Overall, there were 134/150 (89.3%) children that had HCoV IgG test result at all these five timepoints and where included in analysis. Of the mothers, a total of 144/150 (96%) with available plasma were tested for HCoV IgG antibodies. A total of 125/150 children had plasma collected before the pandemic at 6–12 weeks between September 2018 and November 2018 and at 36 months old during the third epidemic waves of the pandemic in Zambia between August 2021 and November 2021 and were included in the SARS-CoV-2 IgG testing (Fig. 2).

### Measurement of HCoV and SARS-CoV-2 specific S1 IgG antibodies

We tested plasma samples using an in-house indirect enzyme-linked immunosorbent assay (ELISA). We used commercially available antigens (Sino Biologicals Inc) for both the endemic HCoV and SARS-CoV-2 ELISA: HCoV-NL63 Spike/S1 Subunit, (40600-V08H), HCoV-OC43 Spike/S1 Protein (40607-V08H1), HCoV-229E Spike/S1 Protein Subunit(40601-V08H), HCoV-HKU1 Spike/S1 Protein Subunit (40021-V08H) and SARS-CoV-2 Spike/S1-His recombinant protein (40591-V08H). Test plasma samples, diluted 1:100, were incubated at 4 °C overnight in duplicate wells of a 96-well microtiter plate (Greiner Bio-One) pre-coated with 0.5 µg/ml HCoV or 1 µg/ml SARS-CoV-2 antigen. Addition of 1:15000 diluted peroxidase conjugated anti-human IgG (Sigma-Aldrich) to the plate and incubation for 3 h at room temperature was used to detect HCoV and SARS-CoV-2 antigen specific antibody responses. ELISA assays were developed by enzymatic reaction with o-Phenylenediamine dihydrochloride substrate (Sigma-Aldrich), development halted by addition of 1 M Sulphuric acid and absorbance measured at a 492 nm wavelength using a microplate reader (Agilent, South Africa). We included adult plasma with known exposure to SARS-CoV-2 and the four endemic HCoV for SARS-CoV-2 and HCoV ELISA respectively. We assigned the SARS-CoV-2 and HCoV positive controls an arbitrary value of 1000 absorbance units in each experiment to generate relative absorbance units (rAU) in the test plasma as concentration readouts.

### Data analysis

Background characteristics were summarized using frequency and proportion for categorical variables while interquartile interval was used for continuous variables. The outcomes of interest were HCoV and SARS-CoV-2 S1 IgG seropositivity. The S1 IgG seropositivity against HCoV-NL63, HCoV-229E, HCoV-OC43 and HCoV-HKU1 was determined using a cut-off calculated by regression finite mixture model of the rAU readouts. The SARS-CoV-2 IgG seropositivity cut-off was calculated as the arithmetic mean rAU of all pre-COVID-19 pandemic samples plus three times the standard deviation. Mann-Whitney U test was used to determine significance between groups. Repeated measures one-way ANOVA and adjustment for multiple comparisons by Tukey’s test was used to assess differences in HCoV IgG antibodies across five timepoints. Spearman *r* was used to evaluate correlations. All analyses were performed in Stata 17 (StataCorp, College Station, TX, USA) and GraphPad Prism v9 (GraphPad Software, LLC). P-values < 0.05 were considered significant and denoted as * (*p* < 0.05), **(*p* < 0.01), ***(*p* < 0.001), ****(*p* < 0.0001) in figures.

## Results

Among the total children with SARS-CoV-2 serostatus available (*n* = 150) median age at baseline was 6 weeks and there no significant differences in general baseline characteristics between children who were SARS-CoV-2 S1 IgG seropositive (*n* = 9) and those that were seronegative (*n* = 141) (Table 1).

**Table 1:** Baseline characteristics of children

Characteristic	*N* (% of Total)	SARS-CoV-2 IgG –ve *n* (%)	SARS-CoV-2 IgG + ve *n* (%)	*p*-value
Total	150 (100.0)	141 (94.0)	9 (6.0)	
Child Age (weeks)				
Median (IQR)	6 (6,6)	6 (6,6)	6 (6,6)	0.771
Sex				
female	66 (44.0)	64 (45.4)	2(22.2)	0.300
male	84 (56.0)	77 (54.6)	7 (77.8)	
Gestation				
Full-term	143 (95.3)	134 (95.0)	9 (100.0)	1.000
Pre-term	7 (4.7)	7 (5.0)	0 (0.0)	
Birth weight, (*n* = 149)				
< 2.5 kg	15 (10.1)	15 (10.7)	0 (0.0)	0.599
≥2.5 kg	134 (89.9)	125 (89.3)	9 (100.0)	
HIV unexposed	103 (68.7)	97 (68.8)	6 (66.7)	1.000
HIV exposed	47 (31.3),	44 (31.2)	3 (33.3),	

### Seroprevalence of endemic HCoV-NL63, HCoV-229E, HCoV-OC43 and HCoV-HKU1

**Fig. 3:**
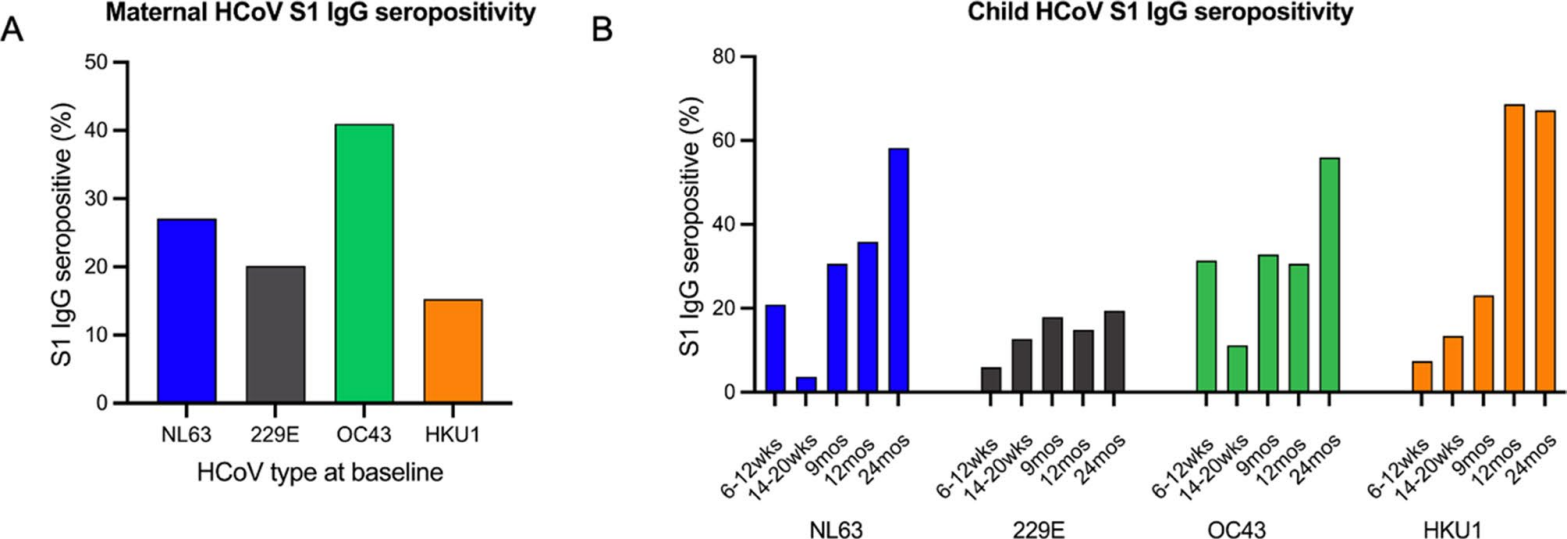
Seroprevalence of endemic HCoV. Percentage of S1 IgG seropositivity against HCoV-NL63, HCoV-229E, HCoV-OC43 and HCoV-HKU1 in mothers at baseline and among children (*n* = 134) at ages 6–12 weeks (baseline), 14–20 weeks, 9 months, 12 months and 24 months. The percentage of S1 IgG seropositive individuals out of total number tested at each timepoint are plotted as bars for each HCoV type

Based on the statistical finite mixture regression models, calculated S1 IgG seropositivity cut-off antibody values among mothers (*n* = 144) were 980.4 rAU for HCoV-NL63, 936.8 rAU for HCoV-229E, 1072.2 rAU for HCoV-OC43 and 945.3 rAU for HCoV-HKU1 (See Supplementary Figure S1, Additional File 1). We restricted analysis of HCoV S1 IgG seropositivity to infants that had available plasma sample at all five timepoints 6–12 weeks, 14–20 weeks, 9, 12 and 24 months (*n* = 134). For these children, the calculated antibody cut-off values for HCoV-NL63, HCoV-229E, HCoV-OC43 and HCoV-HKU1 were 438.2, 856.8, 527.8 and 587.5rAU at ages 6–12 weeks (*n* = 134), 287.7, 256.8, 420.6 and 195.5 rAU at 14–20 weeks (*n* = 134), 66.5, 61.0, 49.3 and 54.7 at 9 months (*n* = 134), 83.4, 74.8, 64.0, and 37.4 rAU at 12 months (*n* = 134) and 108.7, 99.6, 95.3 and 65.1 rAU at 24 months (*n* = 134) respectively (See Supplementary Figure S2, Additional File 2). We found evidence of exposure to the four endemic HCoV species in mothers and children as shown in Fig. 3. Among mothers at baseline, S1 IgG seropositivity was 39/144 (27.1%) for HCoV-NL63, 29/144 (20.1%) for HCoV-229E, 59/144 (41%) for HCoV-OC43 and 22/144 (15.3%) for HCoV-HKU1 (Fig. 3A). The trends in endemic HCoV S1 IgG seropositivity among children at baseline were comparable to that observed in mothers. There were 28/134 (20.9%) children that were S1 IgG positive for HCoV-NL63, 8/134 (5.97%) for HCoV-229E, 42/134 (31.3%) for HCoV-OC43, and 10/134 (7.5%) for HCoV-HKU1. At 14–20 weeks old (*n* = 134), 9 (*n* = 134), 12 (*n* = 134) and 24 months (*n* = 134) old, the S1 IgG seropositivity was 3.7%, 30.6%, 35.8%, and 58.2% for HCoV-NL63, 12.7%, 17.9%, 14.9% and 19.4% for HCoV-229E, 11.2%, 32.8%, 30.6% and 56.0% for HCoV-OC43, and 13.4%, 23.1%, 68.7% and 67.2% for HCoV-HKU1 respectively (Fig. 3B).

### Maternal and child endemic HCoV S1 IgG antibody responses are correlated in early life

**Fig. 4:**
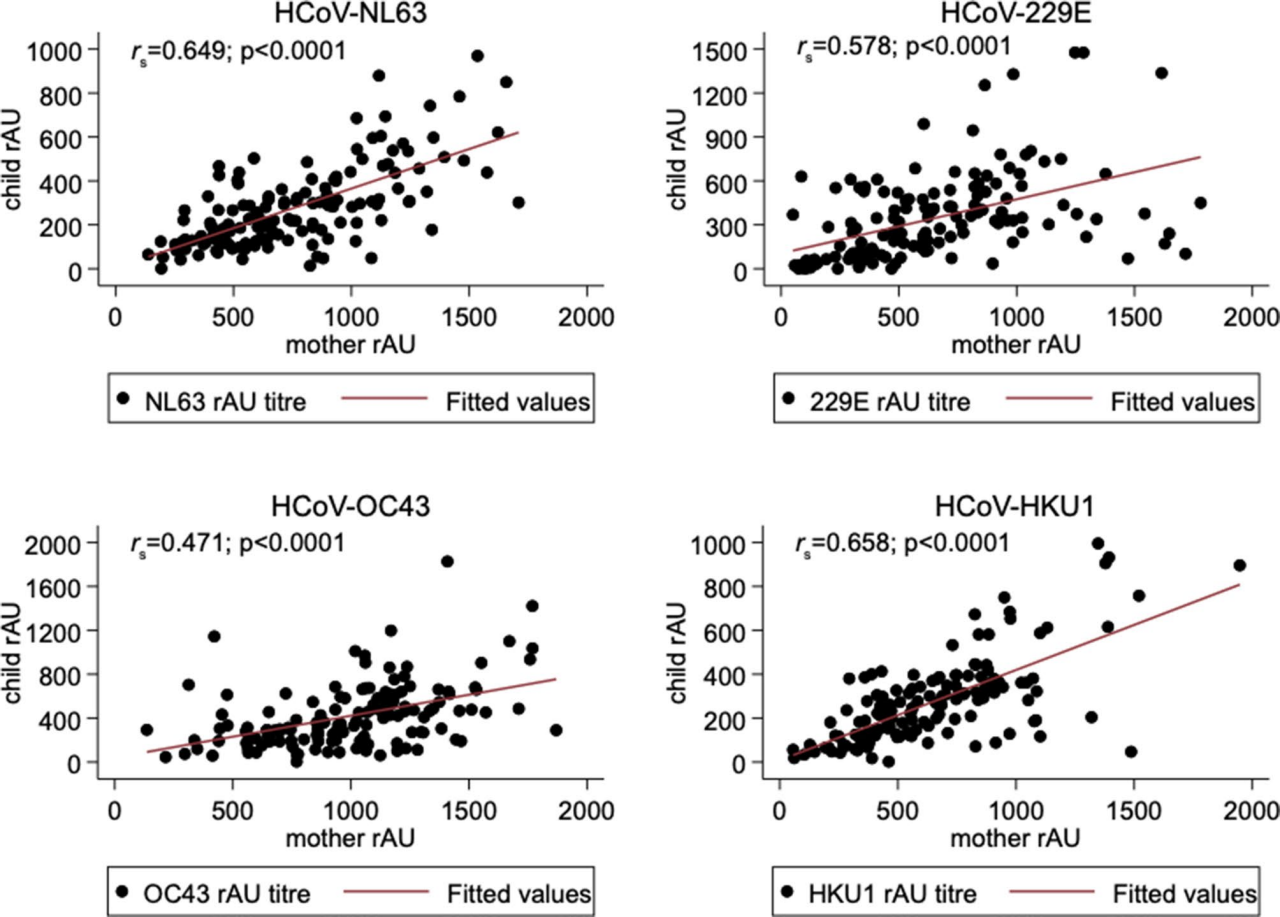
Association of HCoV S1 IgG antibody titres in mothers and children at baseline. Spearman correlation coefficient (*r*_s_) and statistical significance of the relationship between mother and child HCoV-NL63 (**A**), HCoV-229E (**B**), HCoV-OC43 (**C**) and HCoV-HKU1 (**D**) S1 IgG titres at baseline are shown. Each datapoint represents the HCoV specific S1 IgG titre in relative absorbance units in mother-child pairs

We assessed whether the similarities observed between maternal and child HCoV S1 IgG seroprevalence at baseline were reflective of transplacental HCoV S1 IgG transfer. As shown in Fig. 4, a statistically significant relationship between maternal and child (*n* = 142) S1 IgG antibodies at baseline was observed for all four HCoV species with strong to moderate positive correlations for Alphacoronaviruses HCoV-NL63 (Fig. 4A *r*_*s*_=0.649, *p* < 0.0001), HCoV-229E (Fig. 4B *r*_*s*_ =0.578, *p* < 0.0001 *n* = 141) and Betacoronaviruses HCoV-OC43 (Fig. 4C *r*_*s*_ =0.471, *p* < 0.0001) and HCoV-HKU1 (Fig. 4D *r*_*s*_ =0.658, *p* < 0.0001).

### Early seroconversion to all four endemic HCoV in children

**Fig. 5:**
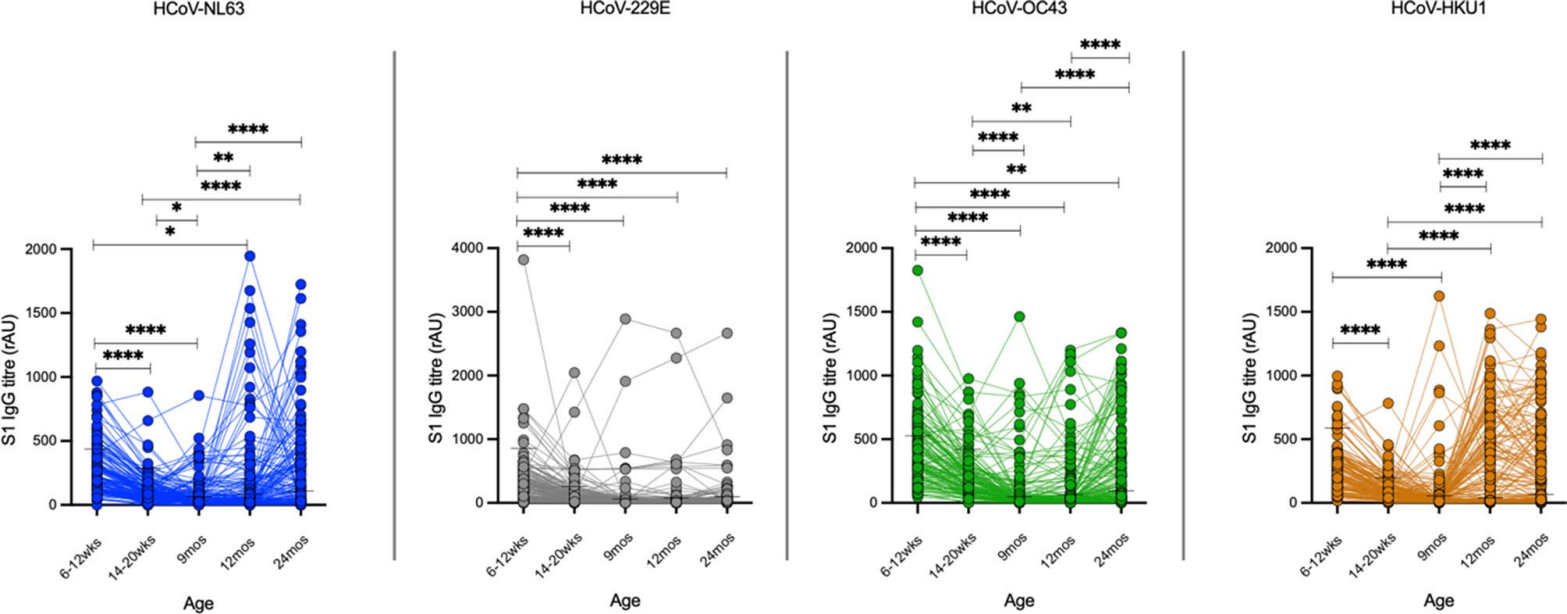
Trajectory of S1 IgG titres against the endemic HCoV in children. Individual level trends of S1 IgG titres against HCoV-NL63 (**A**), HCoV-229E (**B**), HCoV-OC43 (**C**) and HCoV-HKU1 (**D**) in children within the first two years of life. Connected datapoints represent the trajectory of HCoV-specific S1 IgG antibody titres measured in relative absorbance units (rAU) for a single child among children that had data available at all five sampling timepoints (*n* = 134). Horizontal line at each timepoint indicates calculated cut-off value for seropositivity

To investigate the timing of initial seroconversion to the four HCoV in children we studied individual level trends of HCoV specific S1 IgG titres in serially collected plasma limited to children that had data on S1 IgG titres available at all sampling timepoints during the first two years of life (*n* = 134). As shown in Fig. 5, there was a waning of S1 IgG titres observed up to 9 months old for all the four HCoV species (Fig. 5A and D). Significant seroconversion to HCoV-NL63 and HCoV-HKU1 was observed by 12 and 24 months old and HCoV-OC43 seroconversion was also observed by 24 months old. However, there was no significant seroconversion observed for HCoV-229E by 24 months old.

### Association between endemic HCoV and SARS-CoV-2 antibody responses in children

**Fig. 6:**
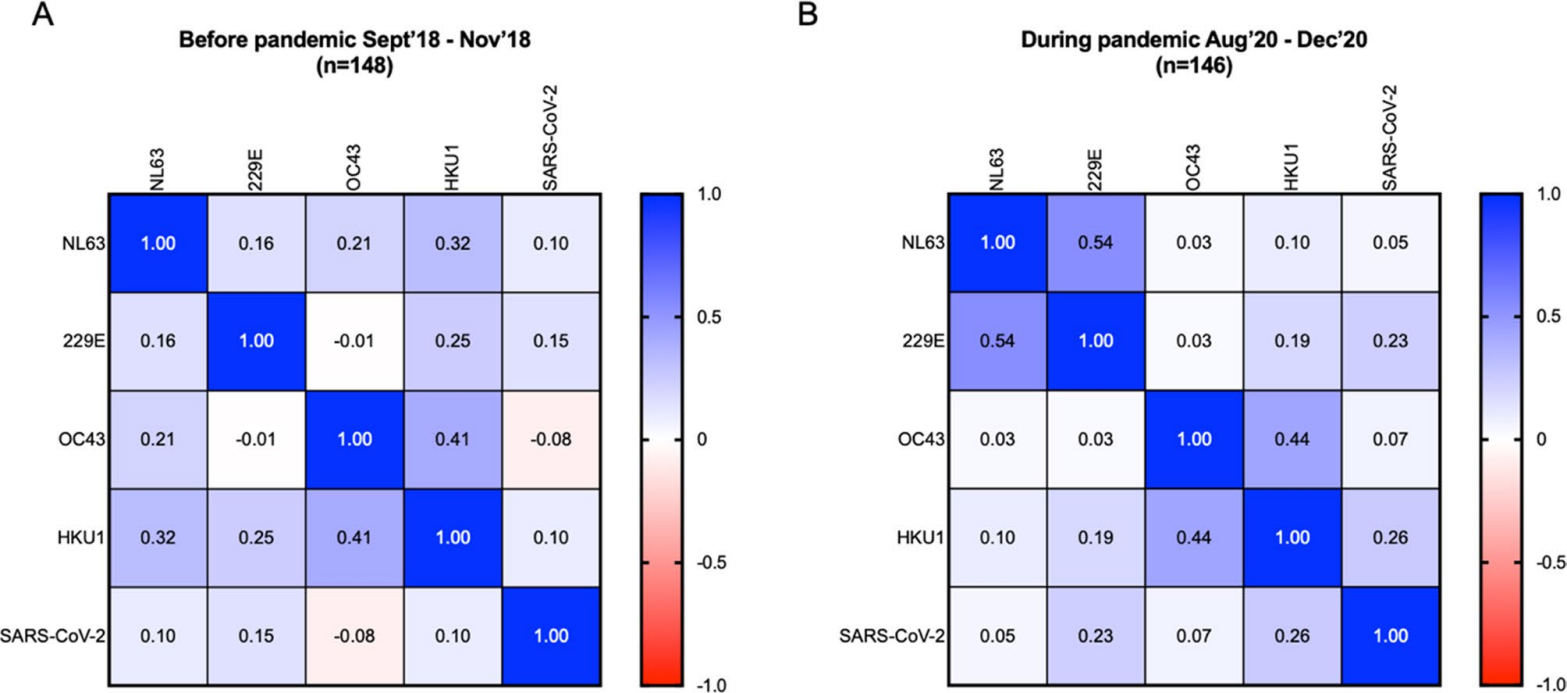
Correlation between HCoV and SARS-CoV-2 S1 IgG titres. Correlation matrix and Spearman correlation coefficients of HCoV and SARS-CoV-2 S1 IgG titres measured in relative absorbance units among children at 6–12 weeks old before the pandemic (**A**, *n* = 148) and at 24 months old during the pandemic (**B**, *n* = 146)

We measured the correlation between endemic HCoV and SARS-CoV-2 S1 IgG antibody titres among children that had results for both endemic HCoV and SARS-CoV-2 S1 IgG at 6–12 weeks old before the pandemic (*n* = 148) and at 24 months during the first wave of the pandemic (*n* = 146). As shown in Fig. 6A, we found no significant correlation between SARS-CoV-2 and endemic HCoV NL63 (*p* = 0.221), 229E (*p* = 0.071), OC43 (*p* = 0.362), and HKU1 (*p* = 0.245) S1 IgG titres and observed weak to moderate correlations within and between the endemic HCoV before the pandemic. During the pandemic as shown in Fig. 6B, SARS-CoV-2 S1 IgG titres were not correlated with endemic HCoV NL63 (*p* = 0.563), and OC43 (*p* = 0.431) but weakly correlated with HCoV 229E (*p* = 0.004) and HKU1 (*p* = 0.002) S1 IgG titres and we observed moderate correlation between HCoV NL63 and 229E (*p* < 0.001) and between HCoV OC43 and HKU1 (*p* < 0.001).

### SARS-CoV-2 S1 IgG seroprevalence in children after the second and third COVID-19 waves

We previously reported SARS-CoV-2 S1 IgG seroprevalence of 9/150 (6%) among the children aged 24 months during the first COVID-19 wave [32]. We tested children with available plasma samples collected at 6–12 weeks pre-pandemic and at 36 months old following the second and third COVID-19 waves (*n* = 125) for SARS-CoV-2 IgG. A cut-off value of 839.6 rAU was calculated using the pre-pandemic plasma. Using this cut-off, a total of 33/125 (26.4%) children were SARS-CoV-2 S1 IgG seropositive at 36 months old.

## Discussion

The seroprevalence data obtained in this study provides evidence that all four endemic HCoV infections are common within our setting. Endemic HCoV prevalence reported in children with acute respiratory illness and among community symptomatic or asymptomatic controls can range below 5% using molecular detection in Zambia [5, 33]. The comparatively higher seroprevalence estimates from our study suggests that most endemic HCoV infections in this population may be asymptomatic or mild and could be underestimated using symptom based testing. Other studies have reported higher likelihood of HCoV detection among asymptomatic children compared to those with respiratory illness in Zambia [6]. We previously reported high incidence of respiratory tract infections within the parent rotavirus vaccine trial [28], however the association between respiratory illness and endemic HCoV seropositivity was not within the scope of this study.

The seroprevalence estimates for specific HCoV species in children in our study differed from those reported in other similar studies conducted among mother-child pairs in China [34, 35] the Netherlands [36] and among children in the Philippines [37] which may be attributed to variation in methodologies used, seasonal HCoV distributions and inclusion of children with acute or severe respiratory illness in the study population. However, our observation of the lowest HCoV immunity by 9 months is consistent with reports noting significant decline in HCoV IgG antibodies or seropositivity within the first year of life [35, 37] most likely reflecting the gradual waning of passive maternal immunity. Like the study by Tomasi et al., we found increasing HCoV spike seropositivity with increasing age [38] but in our study this was for all HCoV species except 229E. This transition from passive to acquired HCoV immunity gives insights for duration of maternal immune protection for HCoV but also for the timing of potential SARS-CoV-2 vaccination in infants should SARS-CoV-2 immunity follow similar trajectories. Global HCoV estimates based on molecular detection report highest prevalence for OC43 in children and HKU1 in adults [39] which contrasted with our results. Most children in our study seroconverted to HCoV by 24 months old and by this age we found HKU1 had the highest seroprevalence in children and seroprevalence was highest for HCoV OC43 among mothers in our study. However, like estimates from global and other studies elsewhere we observed the least seroprevalence and limited seroconversion by 24 months old for 229E compared to other HCoV species in children [35, 39]. Low seroprevalence of HCoV 229E suggests comparatively less circulation of 229E in early life or that passive or induced immunity sufficiently protects against 229E infections or re-infections as reported in some challenge studies [40]. The detection of higher HKU1 in 24 months old children in our setting may have been impacted by our sampling period which aligned with the reported peak for Betacoronaviruses during July and September in Zambia and in which asymptomatic infections are found to be primarily driven by HKU1 [6]. Conversely, low 229E seroprevalence could have been influenced by sampling outside of the reported Alphacoronavirus peak during November to January [6]. Differences in HCoV seasonal variations and temperate versus tropical settings may have factored into the variation in HCoV species seroprevalence observed among children and mothers in this study. For example, a study demonstrated higher breastmilk antibodies to HCoV OC43 in Ugandan compared to American women [41].

During COVID-19 pandemic in Zambia, public health mitigation measures against SARS-CoV-2 in the first epidemic wave were associated with a decline in some respiratory viruses but HCoV infection peaks showed no significant difference before and during the pandemic despite these measures [42]. Markedly higher HCoV peaks were observed in 2020 coinciding with the timing of plasma collection at 24 months in our study and easing of mitigation measures towards end of 2020 [42, 43]. Relaxing of measures may have resulted in continued transmission of HCoV and contributed to the increase in seroprevalence observed among children in our study between 12 and 24 months old for HCoV NL63, OC43 and HKU1. This possibility is exemplified by the resurgence of some respiratory viruses that had previously declined [43]. Nevertheless, interpretations must be made with caution as these HCoV surveillance studies were based on symptomatic individuals and may not have captured the impact of these mitigation measures on the community-level burden accounting for sub-clinical infections.

The lack of correlation between all HCoV species and SARS-CoV-2 S1 IgG in pre-pandemic plasma is consistent with studies reporting evidence of pre-pandemic plasma cross-reactivity significantly against SARS-CoV-2 S2 than S1 subunit [44]. Cross-reactivity of pre-pandemic Zambian samples has been reported predominantly against SARS-CoV-2 nucleocapsid than spike protein [26]. We observed only weak correlations with SARS-CoV-2 S1 IgG for HCoV HKU1 and HCoV 229E in plasma collected during the first COVID-19 wave. Higher HCoV-229E IgG antibodies associated with increased SARS-CoV-2 IgG antibodies are reported by others [45] but some studies have also shown no correlation between HKU1 antibodies and SARS-CoV-2 IgG titres [46]. In another study, significant moderate correlation in IgA of Betacoronaviruses HKU1 and OC43 with SARS-CoV-2 S2 have been documented and similar trends observed for IgG whereas no correlations were noted with SARS-CoV-2 S1 for both IgA and IgG [38]. While our results may indicate an S1 specific back boosting effect of SARS-CoV-2 on HCoV HKU1 and 229E, it is possible that other factors affecting infant’s susceptibility to both these endemic HCoV species and SARS-CoV-2 not included in our analysis may have played a role in these correlations. Nevertheless, our data lends some support for use of S1 based assays for SARS-CoV-2 serological surveillance for enhanced specificity within our setting.

We had previously estimated a seroprevalence of 6% in children below 5 years old during the first COVID-19 wave which was within similar ranges reported by a larger study in Zambia by Mulenga et al. and global estimates among children including this age group [17, 47]. In this study we observed an increase in the SARS-CoV-2 S1 IgG seroprevalence in children to 26.4% by November 2021 consistent with ranges reported from the African region [17] and demonstrating active viral transmission within this population during the third COVID-19 wave in Zambia. Similarly in neighboring Tanzania for example, a rise in SARS-CoV-2 IgG seroprevalence between April to August 2021 was observed among children [48]. These findings support the sharp increase in SARS-CoV-2 seroprevalence observed in Africa during the second and third COVID-19 waves [49] and highlight the wide circulation of SARS-CoV-2 among children within the community.

Key strengths of our study included our longitudinal design spanning both pre- and during first, second and third COVID-19 epidemic waves which was advantageous for temporal profiling of both the HCoV and SARS-CoV-2 antibody responses and exploring correlations. By measuring immunity to individual HCoV species we provide more granularity to HCoV seroprevalence estimates than the use of aggregated HCoV estimates. Our sampling of mother-child pairs also permitted investigation of the transition from passive to acquired immunity for individual endemic HCoV species. This study had limitations. Maternally derived IgG is generally thought to wane within a year after birth, however, the HCoV IgG seropositivity measured at 12 months of age may have included persistent maternal IgG and thus may have not reflected actual child HCoV exposure in some infants. HCoV IgG peaks later than IgM or IgA and we may not have detected individuals with more recent infection [18]. We could not confirm HCoV or SARS-CoV-2 infection by molecular detection or determine the clinical significance of HCoV seropositivity. The HCoV seroprevalence estimates were based on samples collected at timepoints defined under the parent study aims rather than systematically covering all seasons within a calendar year which could have missed known HCoV transmission periods [6, 43] and biased our estimates.

## Conclusions

There is wide circulation of endemic HCoV among adults and children in Zambia with exposure occurring in the first year of life after waning of maternal immunity. In the post COVID-19 era, SARS-CoV-2 immunity among mother-child dyads may follow similar patterns which can provide insights for control measures in early life such as maternal SARS-CoV-2 vaccination. Children are susceptible to SARS-CoV-2 infection and induced immunity in this sub-population can contribute to herd protection in Zambia. This highlights the need for continued SARS-CoV-2 surveillance and consideration of SARS-CoV-2 vaccine strategies in children.

## Supplementary Information

Below is the link to the electronic supplementary material.

Supplementary Material 1: Additional File 1 Image (PNG). Maternal HCoV finite mixture model plots. Supplementary Figure S1. Cut-off values for maternal coronavirus specific rAU titres calculated from finite mixture regression models. The predicted normal distributions of seronegative (red) and seropositive (green) populations are shown overlayed on histogram plots of the spike S1 IgG antibody titres for HCoV-NL63, HCoV-229E, HCoV-OC43 and HCoV-HKU1 among mothers (n = 144) at baseline. The titre cut-off value (vertical dashed line) was calculated as the mean of the seronegative population plus 3x the standard deviation for each HCoV type

Supplementary Material 2: Additional File 2 Image (PNG). Child HCoV finite mixture model plots. Supplementary Figure S2. Cut-off values for child HCoV specific rAU titres calculated from finite mixture regression models. The predicted normal distributions of seronegative (red) and seropositive (green) populations are shown overlayed on histogram plots of the spike S1 IgG antibody titres for HCoV-NL63, HCoV-229E, HCoV-OC43 and HCoV-HKU1 among children aged 6–12 weeks (n = 134) 14–20 weeks (n = 134), 9 months (n = 134), 12 months (n = 134) and 24 months (n = 134). The titre cut-off value (vertical dashed line) was calculated as the mean of the seronegative population plus three times the standard deviation for each HCoV type at each timepoint

## Data Availability

The datasets used and/or analysed during the current study are available from the corresponding author on reasonable request.

## Abbreviations

COVID-19: Coronavirus disease 2019
ELISA: Enzyme-linked immunosorbent assay
HCoV: Human coronavirus
HIV: Human immunodeficiency virus
IgA: Immunoglobulin A
IgG: Immunoglobulin G
IgM: Immunoglobulin M
MERS-CoV: Middle eastern respiratory syndrome coronavirus
rAU: Relative absorbance units
S: Spike
S1: Spike subunit 1
S2: Spike subunit 2
SARS-CoV: Severe acute respiratory syndrome coronavirus
SARS-CoV-2: Severe acute respiratory syndrome coronavirus 2
