# Supplementary figures and images for: A longitudinal study of antibody responses to endemic HCoV and novel SARS-CoV-2 among mother-child pairs in Zambia
Source: BMC Infect Dis. 2025 Nov 18;25:1610. doi: 10.1186/s12879-025-11974-4 (PMC12625463; doi:10.1186/s12879-025-11974-4)

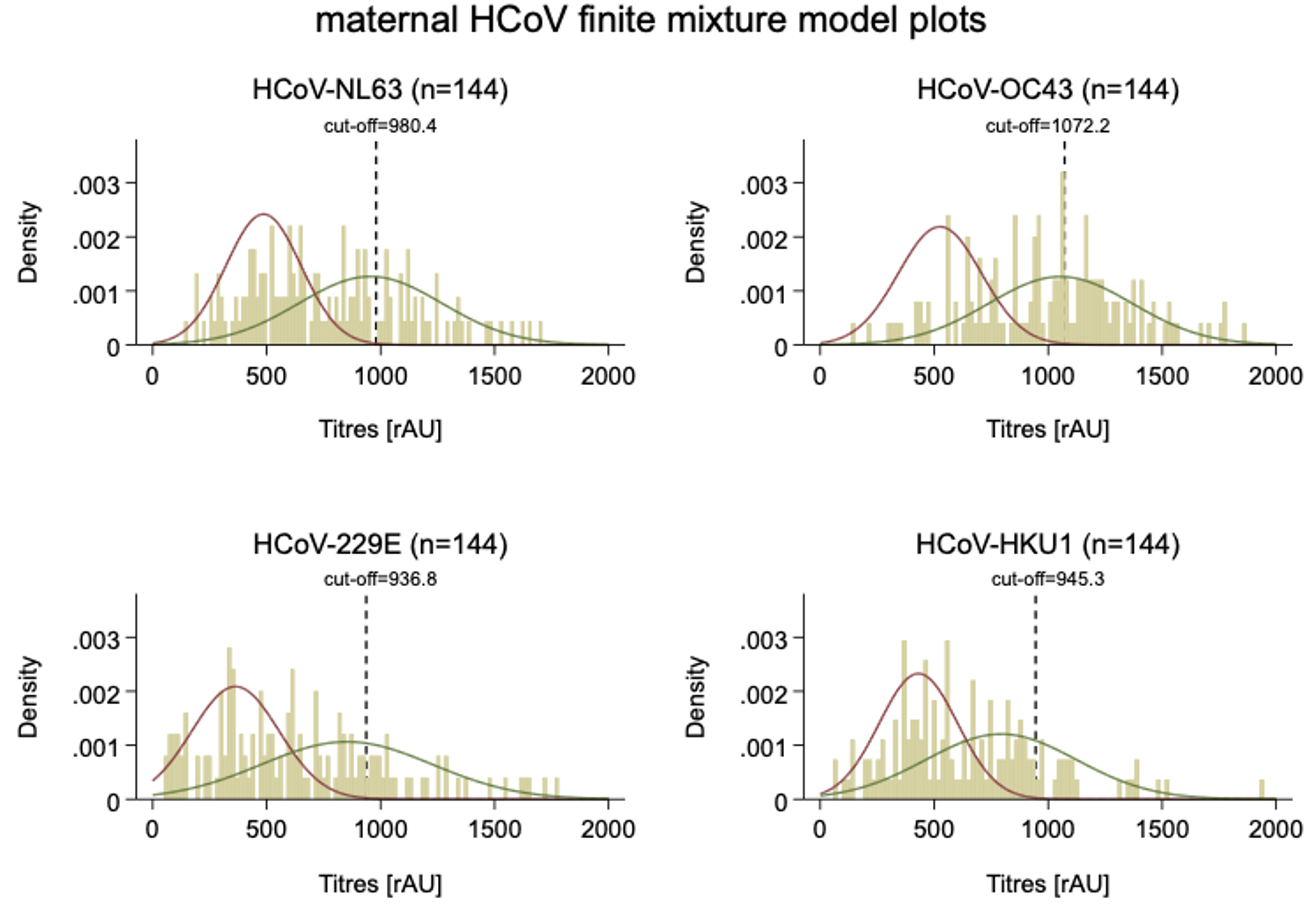

Supplement: Supplementary file 1 — Supplementary Material 1: Additional File 1 Image (PNG). Maternal HCoV finite mixture model plots. Supplementary Figure S1. Cut-off values for maternal coronavirus specific rAU titres calculated from finite mixture regression models. The predicted normal distributions of seronegative (red) and seropositive (green) populations are shown overlayed on histogram plots of the spike S1 IgG antibody titres for HCoV-NL63, HCoV-229E, HCoV-OC43 and HCoV-HKU1 among mothers (n = 144) at baseline. The titre cut-off value (vertical dashed line) was calculated as the mean of the seronegative population plus 3x the standard deviation for each HCoV type [file 12879_2025_11974_MOESM1_ESM.png]

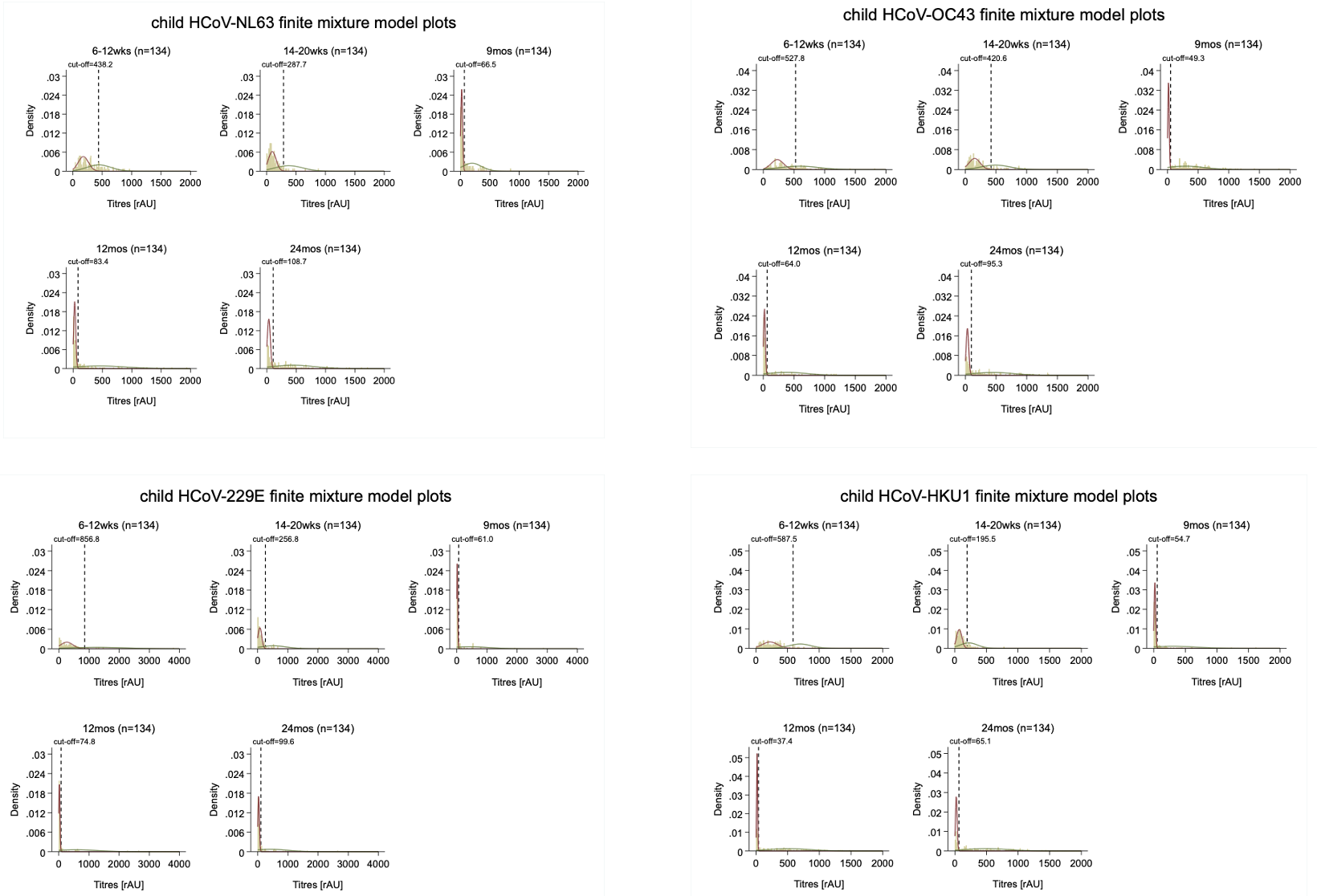

Supplement: Supplementary file 2 — Supplementary Material 2: Additional File 2 Image (PNG). Child HCoV finite mixture model plots. Supplementary Figure S2. Cut-off values for child HCoV specific rAU titres calculated from finite mixture regression models. The predicted normal distributions of seronegative (red) and seropositive (green) populations are shown overlayed on histogram plots of the spike S1 IgG antibody titres for HCoV-NL63, HCoV-229E, HCoV-OC43 and HCoV-HKU1 among children aged 6–12 weeks (n = 134) 14–20 weeks (n = 134), 9 months (n = 134), 12 months (n = 134) and 24 months (n = 134). The titre cut-off value (vertical dashed line) was calculated as the mean of the seronegative population plus three times the standard deviation for each HCoV type at each timepoint [file 12879_2025_11974_MOESM2_ESM.png]
